# Three-Dimensional Finite Element Analysis of Maxillary Protraction Using Diverse Modes of Rapid Palatal Expansion

**DOI:** 10.7759/cureus.36328

**Published:** 2023-03-18

**Authors:** Rajkumar Balakrishnan, Nagalakshmi Sengottuvel, Syed Khalid Altaf, Pawan Kumar Bhandari, Preethi Kumaragurubaran, Marshal Antony

**Affiliations:** 1 Department of Orthodontics and Dentofacial Orthopedics, Vivekanandha Dental College for Women, Tiruchengode, IND

**Keywords:** malocclusion, hygenic rapid expander, rapid palatal expanders, maxillary protraction, finite element analysis

## Abstract

Introduction:Three-dimensional finite elemental analysis (FEA) is a contemporary research instrument for the numeric simulation of a real physical system’s mechanical process. FEA can be used as a very effective tool to analyze and compare various aspects of rapid palatal expanders and to determine the stress distribution in maxillofacial bones and displacement and the biomechanical effects it has on the circummaxillary sutures. This study evaluates the effects of different modes of rapid palatal expansion on maxillary protraction as a treatment modality in skeletal Class III malocclusion by determining the stress and displacement along the circummaxillary sutures using the FEA.

Materials and methods: Initially, a three-dimensional finite element simulation of the maxillofacial skeleton and sutures was obtained by Mimics software (Leuven, Belgium) from the cone-beam computed tomography (Dentsply Sirona, USA) images of a 30-year-old adult with normal occlusion. A geometrical preparation of the three expansion appliances, (A) hybrid MARPE (miniscrew-assisted rapid palatal expander)* *appliance (Fav anchor, India), (B) tooth-borne HYRAX (hygenic rapid expander) appliance (Welcare orthodontics, Kerela), and (C) bone-borne modified MARPE appliance (Biomaterials, Korea), was transferred to ANSYS WORKBENCH, 2020 R1 software (ANSYS, Inc., USA), and three finite element models with each appliance were prepared. A protraction force of 500g was applied to the occlusal plane that is directed 20 degrees inferiorly. The tensile stress, compressive stress, and the amount of displacement on the circummaxillary sutures were assessed and compared in all the three appliances. Young’s modulus (kg/mm^2^) and Poisson’s ratio (V) were used to calculate the stress and displacement in sutures adjacent to the maxilla in different aspects.

Results: On analyzing the stress distribution, the tensile stress was found to be maximum in the medial aspect of the frontomaxillary suture of the bone-borne modified MARPE appliance (C), and the minimum tensile stress was found in the lateral aspect of the sphenozygomatic suture in hybrid MARPE (A). Again, the compressive stress distribution was found to be maximum in the medial aspect of the frontomaxillary suture in all three simulations and the minimum compressive stress in the superior aspect of the internasal suture in hybrid MARPE (A) along with the frontonasal suture at its medial aspect for tooth-borne HYRAX (B) and bone-borne modified MARPE (C). Displacement of the maxilla in all the planes was observed to be the largest for the bone-borne modified MARPE (C) appliance. On the contrary, the minimum displacement was found in the tooth-borne HYRAX (B) appliance.

Conclusion: The findings reveal that all three modes of rapid palatal expanders produced stress and displacement along the circummaxillary sutures on the application of protraction force with bone-borne modified MARPE being more effective in treating posterior crossbites thereby correcting the skeletal Class III malocclusions successfully.

## Introduction

Maxillary protraction with face mask therapy is a significant treatment modality for skeletal Class III malocclusion with maxillary deficiency at growing age. The protractive force should be applied to the maxilla as a single unit for it to be effective [1]. The extra-oral appliance is the source from which the intra-oral appliance gives force to the maxilla. The circummaxillary articulations are affected by palatal expansion apart from the expansion that disarticulates the maxillary bone. A more positive change to the protraction forces is evident with this cellular response [2]. The maxilla and maxillary dentition thus rotate in a forward and downward direction [3].

One of the effective ways to treat transverse deficiency in Class III malocclusion is rapid palatal expansion (RPE) [3]. The forces produced by the rapid palatal expander are transmitted to the palatal vault along the teeth and alveolar process of the maxilla. These forces disarticulate maxillary sutures; thus the mid-palatal suture disengages, and the skeletal expansion starts [4]. The commonly used rapid palatal expanders are tooth-borne conventional RPE, bone-borne RPE, and hybrid RPE.

Various studies have assessed and compared these rapid palatal expanders and their effects on the circummaxillary sutures [1, 3, 5, 6]. Three-dimensional finite elemental analysis (FEA) is a contemporary research instrument for the numeric simulation of a real physical system’s mechanical process [7]. The FEA can be used as a very effective tool to analyze and compare various aspects of rapid palatal expanders and determine the stress distribution, displacement, and biomechanical effects it has on the circummaxillary sutures [3, 8-10]. This study evaluates the effectiveness of different modes of RPE on maxillary protraction by determining the stress and displacement along the circummaxillary sutures using the FEA.

## Materials and methods

First, a three-dimensional finite element simulation of the maxillofacial skeleton and a physical model of the sutures were established. For that, a cone beam computerized tomography of an adult of 30 years with normal occlusion and no temporomandibular disease was chosen. Finally, a three-dimensional finite element model of the maxillofacial skeleton and sutures was obtained by Mimics software (Leuven, Belgium).

Three-dimensional coordinate axis distribution as well as displacement in the maxillary sutures

X denotes the transverse plane, Y denotes the anteroposterior plane, and Z the vertical plane. The positive values on these planes show forward, outward, and downward displacements of the maxilla. The physical properties of the materials used are shown below (Table 1) [11].

**Table 1:** The physical properties of the materials used

	Young’s modulus (kg/mm^2 ^)	Poisson’s ratio (V)
Cancellous bone	137	0.3
Compact bone	1370	0.3
Suture	0.7	0.4
Teeth	2070	0.3

*Design of the RPE Appliances*: In the study, three diverse modes of rapid palatal expanders are used: hybrid MARPE (miniscrew-assisted rapid palatal expander) appliance (Fav anchor, India), tooth-borne HYRAX (hygenic rapid expander) appliance (Welcare orthodontics, Kerela), and bone-borne modified MARPE appliance (Biomaterials, Korea). The rapid palatal appliance was designed in such a way as to open the palate in the sutural region at a rate of 1mm per side.

*Hybrid MARPE Appliance (Simulation A)*: The MARPE appliance includes a HYRAX screw with the extension attached to the first molars and first premolars. The bands for molars are meshed with shell elements. A tied interface connects it to the teeth and is then attached with a 0.9mm stainless steel wire on the palatal sides [7].

*Tooth-Borne RPE Appliance (Simulation B)*: The tooth-borne expander used in this study is HYRAX type. Here, bands are attached to the premolars and first molars. Two-dimensional images of the appliance are used to simulate the first model as tooth-borne RPE [12].

*Bone-Borne or Modified MARPE Appliance (Simulation C)*: A two-dimensional Micro-4 HYRAX appliance, with four palatal miniscrews and the arms of the HYRAX screw bent to fit into the collar design, was used for the bone-borne appliance to simulate the three-dimensional finite element model for bone-borne rapid palatal expander [13].

After the completion of the design of the rapid palatal expanders, a geometrical preparation of the three appliances, (A) hybrid MARPE appliance, (B) tooth-borne HYRAX appliance, and (C) bone-borne modified MARPE appliance, using Auto CAD (computer-assisted design, Simens NX CAD, India) and CREO (PTC CREO PARAMETRIC 7.0 M010, Weikfield IT Citi Info Park, Maharashtra) software was done. All these components were transferred to ANSYS WORKBENCH, 2020 R1 software (ANSYS, Inc., USA), and three finite element models with each appliance were prepared. A protractive force of 500g was directed 20 degrees below the plane of occlusion. That means the force is directed around the Z axis at approximately -20 degrees inferiorly in all three models, according to Oppenheim [14]. The three-dimensional finite element model of the maxillofacial skeleton and sutures was prepared using 170012 nodes and 86156 elements for hybrid MARPE (A), 154807 nodes and 83636 elements for tooth-borne HYRAX (B), and 172946 nodes and 95745 elements for bone-borne modified MARPE (C) appliance. Young’s modulus (kg/mm^2^) and Poisson’s ratio (V) were used to calculate the stress and displacement in sutures adjacent to the maxilla in different aspects [15].

The tensile stress, compressive stress, and the amount of displacement on the circummaxillary sutures were assessed and compared in all the three appliances, hybrid MARPE (A), tooth-borne HYRAX (B), and bone-borne modified MARPE (C).

The stress distribution was assessed and compared in the following circummaxillary sutures: (i) frontomaxillary (medial and lateral), (ii) nasomaxillary (superior and inferior), (iii) zygomaticomaxillary (superior, middle, and inferior), (iv) zygomaticotemporal (superior, middle, and inferior), (v) zygomaticofrontal (antero-lateral, antero-medial, postero-lateral, and postero-medial), (vi) frontonasal (medial and lateral), (vii) internasal (inferior and superior), (viii) sphenozygomatic (lateral and medial), and (ix) pterygomaxillary sutures (inferior and superior).

The amount of three-dimensional displacement was measured using the ANSYS software at the points: (i) anterior nasal spine (ANS), (ii) point A, (iii) prosthion (Ps), and (iv) posterior nasal spine (PNS) [14].

## Results

The three RPE appliances (i) hybrid MARPE (A), (ii) tooth-borne HYRAX (B), and (iii) bone-borne modified MARPE (C) were evaluated for the stress distribution at each suture and the displacement to the forces applied.

Comparison of the tensile stress distribution produced by hybrid MARPE (A), tooth-borne HYRAX (B), and bone-borne modified MARPE (C)

On analyzing the tensile stress distribution, it was found to be maximum in the medial aspect of the frontomaxillary suture for bone-borne modified MARPE appliance (C) and the minimum tensile stress was found in the lateral aspect of sphenozygomatic suture in hybrid MARPE (A). In tooth-borne HYRAX (B) and bone-borne modified MARPE (C) appliances, the stresses appeared to be that of a wide range in the medial aspect of the frontomaxillary suture, and it got narrower in the medial aspect of the sphenozygomatic suture (Table 2).

**Table 2:** Maximum tensile stress distribution between hybrid MARPE, tooth-borne HYRAX, and bone-borne modified MARPE at the sutures adjacent to the maxilla with maxillary protraction and expansion (kg/mm2) Mpa, megapascal; MARPE, miniscrew-assisted rapid palatal expander; HYRAX, hygenic rapid expander

Sl no.	Sutures				Hybrid MARPE (Mpa)	Tooth-borne HYRAX (Mpa)	Bone-borne modified MARPE (Mpa)
1	Frontomaxillary suture	Medial			11.016	13.91	17.832
		Lateral			10.18002	11.923	14.516
2	Nasomaxillary suture	inferior			2.0588	2.1659	2.2054
		Superior			1.9845	2.6495	2.7354
3	Zygomaticomaxillary suture	Superior aspect	Antero-medial		1.0546	1.12641	1.3645
			Postero-lateral		1.6243	1.7321	1.6532
		Middle aspect	Antero-medial		1.9846	2.2563	2.3564
			Postero-lateral		2.1562	2.0654	2.2564
		Inferior aspect	Antero-medial		2.0154	2.2468	2.1046
			Postero-lateral		2.1456	2.5491	2.6431
4	Zygomaticotemporal suture	Superior aspect		Lateral	1.1632	1.25465	1.0467
				Medial	1.5623	2.6456	2.45672
		Middle aspect		Lateral	1.6495	1.4525	1.5421
				Medial	1.6879	1.9741	2.214
		Inferior aspect		Lateral	1.4576	1.4941	1.5321
				Medial	1.6465	1.7412	1.9456
5	Zygomaticofrontal suture	Antero-lateral			0.2473	1.3456	1.4512
		Antero-medial			1.5455	1.4215	1.7456
		Postero-lateral			1.6431	1.8453	1.9145
		Postero-medial			1.8876	1.9785	2.3546
6	Frontonasal suture	Medial			0.2473	1.3456	1.4512
		Lateral			0.2377	1.12456	1.3487
7	Internasal suture	Inferior			0.1753	2.0245	2.2478
		Superior			0.17706	1.2456	1.3695
8	Sphenozygomatic suture	Lateral			0.5240	1.1654	2.2456
		Medial			0.5465	0.7896	0.4564
9	Pterygomaxillary suture	Inferior			1.9862	2.0875	2.1245
		Superior			1.8946	2.1564	2.2465

Comparison of the compressive stress distribution between hybrid MARPE (A), tooth-borne HYRAX (B), and bone-borne modified MARPE (C)

On analyzing the appliances, the compressive stress was found to be highest in the medial aspect of the frontomaxillary suture in all three simulations, a minimum stress distribution was observed in the superior aspect of the internasal suture in hybrid MARPE (A), and for the frontonasal suture, it appears medially with the tooth-borne HYRAX (B) as well as bone-borne modified MARPE (C) (Table 3).

**Table 3:** Maximum compressive stress distribution between hybrid MARPE, Tooth-borne HYRAX, and bone-borne modified MARPE at the sutures adjacent to the maxilla with maxillary protraction and expansion (kg/mm2) Mpa, megapascal; MARPE, miniscrew-assisted rapid palatal expander; HYRAX, hygenic rapid expander

Sl no.	Sutures				Hybrid MARPE (Mpa)	Tooth-borne HYRAX (Mpa)	Bone-borne modified MARPE (Mpa)
1	Frontomaxillary suture	Medial			13.255	17.885	21.148
		Lateral			12.563	15.899	20.166
2	Nasomaxillary suture	inferior			4.2981	4.3529	4.4256
		Superior			4.1263	4.2466	4.3156
3	Zygomaticomaxillary suture	Superior aspect	Antero-medial		1.5072	1.4035	1.5325
			Postero-lateral		1.6396	1.7156	1.8256
		Middle aspect	Antero-medial		2.0154	2.1954	2.5649
			Postero-lateral		2.3294	2.4864	2.735
		Inferior aspect	Antero-medial		2.1646	2.2564	2.2413
			Postero-lateral		2.1896	2.4871	2.7451
4	Zygomaticotemporal suture	Superior aspect		Lateral	1.9875	2.0789	2.1458
				Medial	1.6482	1.8451	1.1024
		Middle aspect		Lateral	1.9864	2.0458	2.1458
				Medial	1.9762	2.1458	2.3432
		Inferior aspect		Lateral	1.6594	1.7345	1.9562
				Medial	1.9765	2.2468	1.9458
5	Zygomaticofrontal suture	Antero-lateral			1.4682	1.6458	2.2456
		Antero-medial			1.6582	1.7256	2.2145
		Postero-lateral			1.5666	1.6254	1.8245
		Postero-medial			1.8462	1.9781	2.0645
6	Frontonasal suture	Medial			0.3283	0.1245	0.01456
		Lateral			0.333	0.565	0.789
7	Internasal suture	Inferior			0.18506	1.2456	1.3455
		Superior			0.18268	2.1456	2.2463
8	Sphenozygomatic suture	Lateral			0.6894	1.7845	1.9456
		Medial			0.6943	1.0458	1.1356
9	Pterygomaxillary suture	Inferior			1.6496	2.8456	2.2145
		Superior			1.9746	2.1456	2.01456

Comparison of the range of displacement at the sutures between hybrid MARPE (A), tooth-borne HYRAX (B), and bone-borne modified MARPE (C)

The displacement was calculated and determined separately with the help of reference points of ANS, PNS, Ps, and point A on the three axes X, Y, and Z. The transverse (X-axis), antero-posterior (Y-axis), and vertical (Z-axis) displacements of the maxilla are the largest for bone-borne modified MARPE (C) appliance when compared to hybrid MARPE (A) and tooth-borne HYRAX (B) appliance. On the contrary, the minimum displacement was found in the tooth-borne HYRAX (B) appliance (Table 4).

**Table 4:** The amount of displacement at sutures adjacent to maxilla on comparing hybrid MARPE (A), tooth-borne HYRAX (B), and bone-borne modified MARPE (C) ANS, anterior nasal spine; PNS, posterior nasal spine; Ps, prosthion; MARPE, miniscrew-assisted rapid palatal expander; HYRAX, Hygenic rapid expander

Displacement	Hybrid MARPE (A)				Tooth-borne HYRAX (B)				Bone-borne modified MARPE (C)			
	X-axis	Y-axis	Z-axis	XYZ	X-axis	Y-axis	Z-axis	XYZ	X-axis	Y-axis	Z-axis	XYZ
	mm	mm	mm	Mm	mm	mm	mm	mm	mm	mm	mm	mm
ANS	-0.3601	-0.0979	-0.1328	0.3961	-0.1741	-0.0855	-0.2293	0.3003	-0.6322	-0.1985	-0.1417	0.6776
A-POINT	-0.4153	-0.0867	-0.1422	0.4474	-0.2262	-0.0773	-0.2371	0.3367	-0.7551	-0.1883	-0.1455	0.7917
Ps	-0.4794	-0.0712	-0.1504	0.5075	-0.2842	-0.0637	-0.2448	0.3805	-0.8943	-0.1794	-0.1447	0.9235
PNS	-0.01	0.0035	-0.0001	0.0106	0.0001	0.0045	-0.0098	0.0108	0.5042	-0.2663	-0.1593	0.592
Total body	0.5902	0.2037	0.2261	0.6049	0.4455	0.1303	0.0892	0.5557	1.143	0.3739	0.3537	1.1562

Among the three appliances, the bone-borne modified MARPE appliance (C) has the largest displacement of 1.16mm, whereas the tooth-borne HYRAX appliance (B) has the least displacement of 0.5557mm. Also, the largest amount of displacement was found to be at point Ps of bone-borne modified MARPE appliance, and the least amount of displacement was found to be at point PNS for hybrid MARPE appliance (A). However, the amount of displacement at point PNS for hybrid MARPE (A) and tooth-borne HYRAX (B) is coinciding.

## Discussion

The management modalities and time of treatment in skeletal Class III malocclusions have remained controversial among clinicians. These are mostly related to the factors such as skeletal dental discrepancies, age, and residual growth. The transverse deficiency should be corrected as soon as it is diagnosed since it is mandatory for establishing normal occlusion. Baik analyzed the effects of maxillary advancement in children. After maxillary protraction, the maxilla as well as the maxillary dentition moved forward and downward similar to our study [1].

In a study done by Gautam et al., the application of maxillary protraction forces produced maximum stress at the sphenozygomatic suture followed by the zygomaticomaxillary and zygomaticotemporal sutures [3]. However, the results of our study show that the maximum stress was in the frontomaxillary suture in the bone-borne modified MARPE appliance (C) followed by nasomaxillary and zygomaticomaxillary sutures. Gautam et al. also reported that high stresses were generated in the craniofacial sutures after maxillary protraction with expansion. Our observations were similar to this study. 

Baik evaluated the effect of protraction force on the maxilla at the craniofacial bones [1]. He found that the greatest stress was at the zygomaticomaxillary suture. However, our study showed the greatest stress was at the frontomaxillary suture. Murugan et al. evaluated the displacement and stress distribution and displacement of the fused maxilla and circummaxillary sutures [7]. They used two different sizes of MARPE using FEM. The MARPE type I (12x12mm microscrew) showed more stress distribution and displacement compared with that of the hybrid MARPE appliance used in our study.

RPE remodels the circummaxillary sutures and is effective for maxillary protraction. Hass observed forward and downward tipping of the maxilla along with the inferior and backward rotation of the mandible [16]. FEA is a practical tool to analyze and compare various aspects of rapid palatal expanders and to determine the distribution of stress, displacement of the maxilla, and the biomechanical effects it has on the circummaxillary sutures.

Kambara reported that postero-anterior traction produced changes in the maxillary tuberosity and circummaxillary sutures [17]. This includes the suture opening, sutural connective tissue fibers getting stretched, and also new bone getting deposited along the stretched fibers. This study is quite similar to our findings showing the changes in circummaxillary suture areas on the application of maxillary protraction force. According to Tanne et al., larger compressive stresses were observed around the zygomaticomaxillary, frontozygomatic, and sutures of the frontonasal region [18]. On the contrary, our study shows that the greatest compressive stress was around the frontomaxillary, nasomaxillary, and zygomaticomaxillary sutures.

In a FEM study by Miyasaka-Hiraga et al., in order to find out the effects of the direction of maxillary protraction forces on biomechanical changes in the craniofacial complex, it was observed that a force directed 30 degrees inferiorly produced a stress distribution that was more uniform at the various transverse planes whereas in our study, a 20-degree protraction force, unique to the occlusal plane, produced a non-uniform stress distribution in all three simulations [19]. Hence, force direction is an important factor in controlling the displacement patterns and the associated forward and downward rotation of the maxilla. Hata et al. observed that with maxillary protraction there is a possibility of anterior maxillary constriction [20]. But, in our study, the rapid palatal expander expands the maxillary arch posteriorly, thus correcting the posterior crossbite effectively.

**Figure 1:**
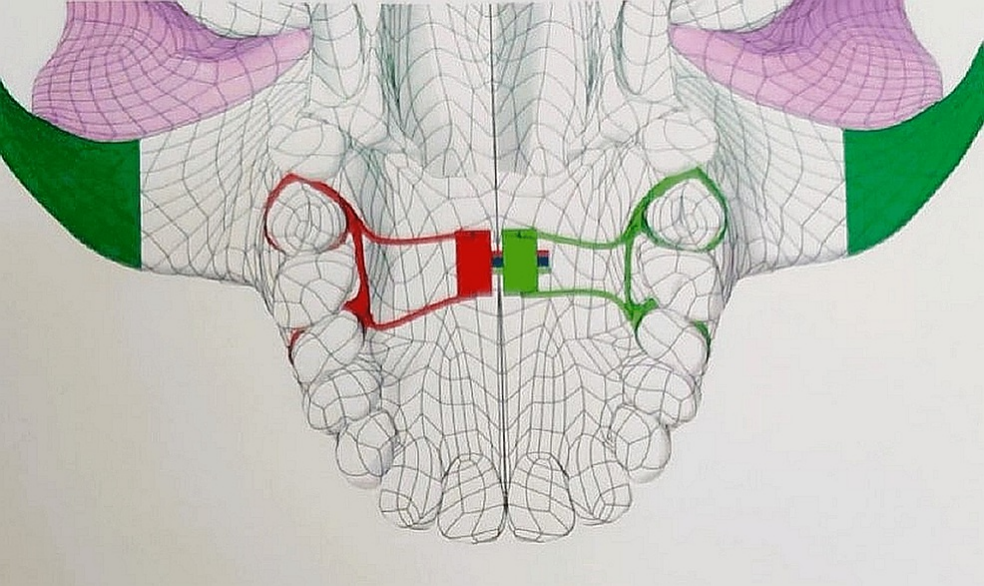
Tooth-borne MARPE simulation model This figure is made by the ANSYS software for testing. MARPE, miniscrew-assisted rapid palatal expander

**Figure 2:**
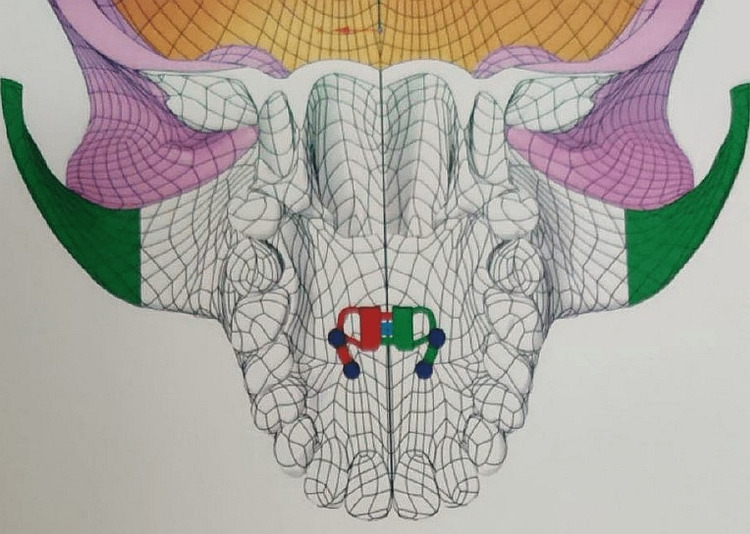
Bone-borne MARPE simulation model This figure is made by the ANSYS software for testing. MARPE, miniscrew-assisted rapid palatal expander

In a study evaluating the distribution of stress and displacement at the craniofacial sutures with rapid maxillary expansion (RME), the maximum tensile stress was found along the frontomaxillary suture, which is similar to our study [21]. Our observations are in agreement with this study, as both point out that high stresses along the different sutures play a very crucial role in the displacement of the maxilla in a forward and downward direction after RPE. Krüsi et al. reviewed the effects of bone-borne or hybrid tooth- and bone-borne RME with conventional tooth-borne RME in maxillary deficiency cases [22]. They observed that bone-borne or hybrid tooth- and bone-borne RME might present advantages in terms of increased sutural opening and reduced tooth tipping. In our study, the bone-borne modified MARPE appliance (C) has the largest displacement, whereas the tooth-borne HYRAX appliance (B) has the least displacement (Figures 1, 2).

The rate of growth of sutures is increased by tension as well as compression, and it is an important factor for the development of the suture. The tensile stress initiates the separation of the cranial sutures and the sutural growth [23]. In a clinical study, Li et al. compare the results of maxillary protraction with and without palatal expansion [24]. He reports that after the application of an advancement force along with the maxilla, the maxillary dentition moved anteriorly with and without expansion. Subsequently, the anterior crossbite was corrected. In contrast, our study shows that maxillary protraction along with RPE produces more stable effects by correcting the posterior crossbite and thus aids in managing skeletal Class III malocclusion more effectively.

The limitation of the study includes that a more advanced study with clinical identification is needed since the impacts on the muscles of the face and other soft tissues also need to be examined.

## Conclusions

In conclusion, the most effective mode of producing RPE among the three appliances used in the study on the application of maxillary protraction force, by using the three-dimensional FEA, is the bone-borne modified MARPE appliance (C) followed by the hybrid MARPE appliance (A) and the least effective being the tooth-borne HYRAX appliance (B) based on the amount of displacement each appliance produces. At the same time, the amount of stress produced was also in a wide range for the bone-borne appliance when compared to the other appliances. The maximum tensile and compressive stresses were produced at the frontomaxillary suture for the bone-borne modified MARPE (C) appliance. The amount of displacement was also greater for the bone-borne modified MARPE appliance (C) followed by the hybrid MARPE appliance (A). However, the tooth-borne HYRAX appliance measured the least amount of displacement.

The findings reveal that all three modes of rapid palatal expanders produced stress and displacement along the circummaxillary sutures on the application of protraction force with bone-borne modified MARPE (C) being more effective in treating posterior crossbites thereby correcting the skeletal Class III malocclusions successfully.
